# Mitochondrial and morphological variation of *Tilapia zillii* in Israel

**DOI:** 10.1186/1756-0500-5-172

**Published:** 2012-04-02

**Authors:** Amir Szitenberg, Menachem Goren, Dorothée Huchon

**Affiliations:** 1 Department of Zoology, Tel-Aviv University, Tel Aviv, 69978, Israel

**Keywords:** *Tilapia zillii*, Population genetics, Mitochondrial control region, Meristic counts

## Abstract

****Background**:**

*Tilapia zillii* is widespread in the East Levant inland aquatic systems as well as in artificial water reservoirs. In this study we explore the genetic and morphological variation of this widespread species, using mitochondrial control region sequences and meristic characters. We examine the hypothesis that *T. zillii'*s population structure corresponds to the four Israeli aquatic systems.

****Results**:**

Out of seven natural water bodies, only two were found to possess genetically divergent populations of *T. zillii*. In addition to its presence in fish farms, the species was found in two artificial recreational ponds which were supposed to have been stocked only with other fish species. In these two artificial habitats, the haplotype frequencies diverged significantly from those of natural populations. Finally, fish from the Dead Sea springs of Ne'ot HaKikar appear to differ both genetically and morphologically from fish of the same aquatic system but not from fish of other water systems.

****Conclusions**:**

Our results show that the population structure of *T. zillii* does not match the geography of the Israeli water-basins, with the exception of the Dead Sea and Kishon River, when considering natural populations only. The absence of a significant divergence between basins is discussed. Our results and observations suggest that the Ne'ot HaKikar Dead Sea population and those of artificial ponds could have originated from the "hitchhiking" of *T. zillii*, at the expense of some other cultivated tilapiine species.

## **Background**

**Figure 1:**
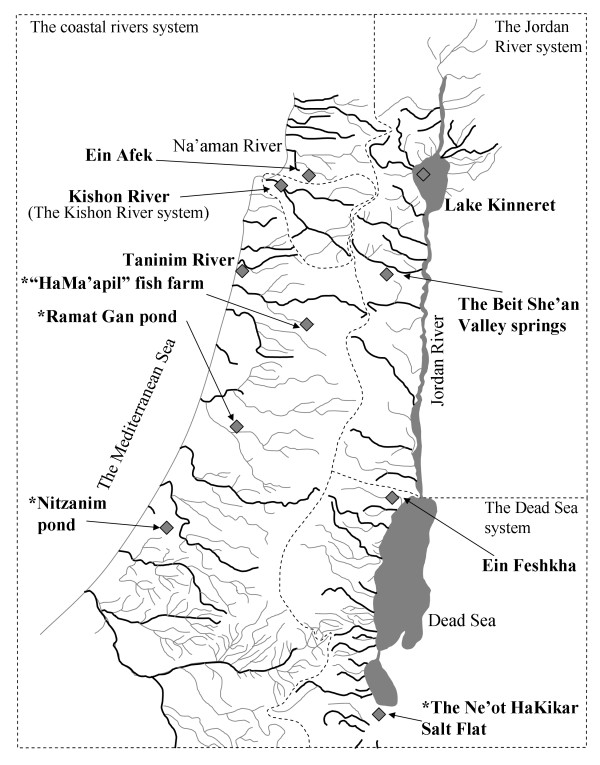
**The inland aquatic systems of Western Levant in the post-glacial period, after Goren & Ortal (1999).** The four aquatic systems are delineated by dashed lines. Populations sampled are in bold and indicated by a gray diamond. Introduced populations are indicated by asterisks.

*Tilapia zillii* (Gervais, 1848) is an African and Middle-Eastern native tilapiine fish [1]. The Israeli populations represent the periphery of Tilapiinae natural geographic distribution. Such peripheral populations may have a high evolutionary significance, as range peripheries are thought to constitute some of the most important areas for speciation [2]. Present-day Israel has three geological aquatic systems (Figure 1): the coastal, the Jordan River, and the Dead Sea systems. Based on faunal composition, a fourth “system”, the Kishon River, can be considered separately [3]. *T. zillii* is present in all four systems. Being euryhaline, *T. zilli* is able to extend its geographic distribution into habitats of a wide salinity range. Specimens have been observed in estuaries and even in shallow marine habitats along the Mediterranean coast [4]. *T. zillii*'s euryhalinity is thus considered to be a key reason for the wide geographic distribution of the species [5,6], in particular in coastal rivers. *T. zillii* is a monogamous biparental guarder and substrate brooder with both parents committed to a single nest through a breeding cycle [7].

Interestingly, *T. zillii* has been found in artificial recreational water reservoirs created in the 1960-70s (e.g., the Ramat-Gan Urban Park and the Nitzanim Nature Reserve, Figure 1). These artificial reservoirs are isolated water bodies that are not connected to any natural habitat. They were stocked with other fish species, in particular tilapiine fishes, from aquacultural supplies. Since *T. zillii* is not listed as a cultivated species in Israel [8], it is not expected to be present in these ponds, which have been populated solely with aquaculture stocks and are isolated from natural water systems. Similarly, in the Dead Sea area, *T. zillii* has been known to be present in the northern springs of the system (e.g., Ein Feshkha, Figure 1) [7]. In the last 20 years the fish has also been detected in the southern section of the Dead Sea system, in the Ne’ot HaKikar area (Figure 1). The origin of these recent populations is unknown. Our study seeks to understand *T. zillii*'s mitochondrial and morphological variations in Israel and its population structure, using the control region sequence and meristic characters as markers.

## **Results**

*T. zillii* was sampled from two coastal system habitats (Ein Afek Nature Reserve and the Taninim River), the Kishon River, two Jordan River habitats (Lake Kinneret and Beit She'an Valley springs) and two Dead Sea system habitats (Ein-Feshkha Nature Reserve and the Ne'ot HaKikar Salt Flat). In addition, two artificial recreational ponds located in the coastal aquatic system were sampled (the Ramat-Gan Park pond and the Nitzanim Nature Reserve pond). All sample sites are indicated in Figure 1.

### **Sequence analysis**

**Figure 2:**
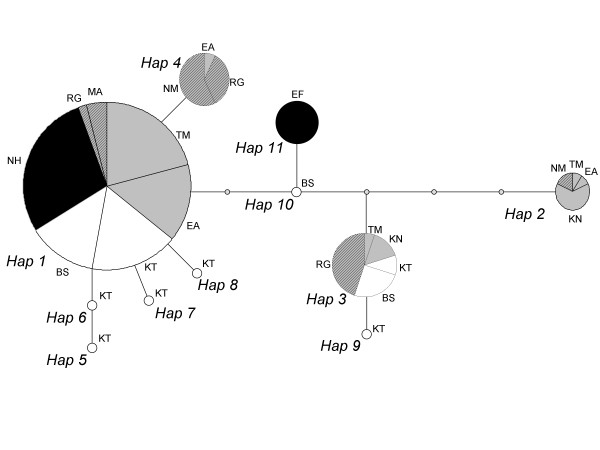
**Median-joining network of*T. zillii*control region sequences**. The sequences are 860-862 bp long and represent Israeli individuals of the following habitats: TM, Taninim (coastal); EA, Ein Afek (coastal); KN, Kishon (Kishon); KT, Kinneret (Jordan); BS, Beit She’an (Jordan); EF, Ein Feshkha (Dead Sea); NH, Ne’ot HaKikar (Dead-Sea introduced); RG, Ramat Gan (coastal introduced); NM, Nitzanim (coastal introduced); MA, HaMa'apil (coastal introduced). The haplotype numbers (in italics) correspond to the numbers in Table 1.

Eleven haplotypes were recognized among the 117 *T. zillii* D-loop sequences obtained (Table 1). The most widespread haplotype (one) was found in all samples except for the Kishon River (Kishon system), Ein Feshkha (Dead Sea sytem), and Nitzanim (coastal introduced) samples. The two fish farm specimens also possessed this haplotype. The Ein Feshkha and Ne'ot HaKikar samples (both from the Dead Sea system) were monomorphic, but for different haplotypes (Table 1; Figure 2). The Kinneret sample (Jordan River system) showed greater haplotype diversity than any other (*h* = 0.69, sd = 0.12). As a consequence of the high haplotype diversity, the effective size estimation of this population is also higher than all the others (*θ*_s_ = 2.11, sd = 1.05), with the Kinneret being the largest most continuous body of water examined. Among the artificial ponds' samples, that from Ramat Gan was the most diversified (*h* = 0.56, sd = 0.09). With the exception of the monomorphic Dead Sea samples, the Kishon River sample provided the smallest *θ*_s_ estimation (*θ*_s_ = 0.35, sd = 0.35) (Table 1).

**Table 1:** **Molecular diversity of*T. zillii*populations**

	**Aquatic system**	**Coastal**					**Kishon**	**Jordan River**		**Dead Sea**	
	Population	Taninim	Ein Afek	Ramat Gan*	Nitzanim*	HaMa'apil*	Kishon	Kinneret	Beit She'an	Ein Feshkha	Ne'ot HaKikar*
Hap.	Polymorphic sites										
	111123357777										
	888882581455										
	234636718807										
1	GTGATAATACA-	11	8	1	0	2	0	9	7	0	15
2	..A..G.C…C	1	1	0	2	0	7	0	0	0	0
3	..A..G…-.-	1	0	9	0	0	3	2	5	0	0
4	......G....-	0	1	5	8	0	0	0	0	0	0
5	A..G.......-	0	0	0	0	0	0	1	0	0	0
6	A..........-	0	0	0	0	0	0	1	0	0	0
7	.C.........-	0	0	0	0	0	0	1	0	0	0
8	..........G-	0	0	0	0	0	0	1	0	0	0
9	..A..G..G-.-	0	0	0	0	0	0	1	0	0	0
10	.......C.-.-	0	0	0	0	0	0	0	1	0	0
11	..A.C..C.-.-	0	0	0	0	0	0	0	0	13	0
	n	13	10	15	10	2	10	16	13	13	15
	*h*	0.29 ±0.16	0.38 ±0.18	0.56 ±0.09	0.35 ±0.16	0	0.47 ±0.13	0.69 ±0.12	0.6 ±0.09	0	0
	*Π*	0.001 ±0.001	0.001 ±0.001	0.002 ±0.001	0.002 ±0.001	0	0.002 ±0.00	0.002 ±0.001	0.002 ±0.001	0	0
	*Ө*_*s*_	0.97 ±0.001	1.41 ±0.86	0.92 ±0.6	1.41 ±0.86	0	0.35 ±0.35	2.11 ±1.05	0.97 ±0.63	0	0
	*Ө*_*π*_	1.03 ±0.82	1 ±0.83	2.02 ±1.35	1.77 ±1.26	0	1.4 ±1.06	1.71 ±1.18	1.72 ±1.2	0	0

From left to right: haplotype number (Hap.), the polymorphic sites in the Control region sequence (polymorphic sites), the number of occurrences of each haplotype in each sample. Diversity parameters : n = sample size, *h* = haplotype diversity, *π* = nucleotide diversity, *Ө*_*s*_ and *Ө*_*π*_ -estimates of population effective size multiplied by the mutation rate. *Introduced population.

**Table 2:** Pairwise population divergence

	**1**	**2**	**3**	**4**	**5**	**6**	**7**	**8**	**9**
1: Taninim (coastal)		<10^-5 ns^	0.44^***^	0.56^***^	1.39^***^	<10^-5 ns^	0.03^ns^	2.58^***^	0.03^***^
2: Ein Afek (coastal)	<10^-5 ns^		0.47^**^	0.41^***^	1.53^***^	<10^-5 ns^	0.09^ns^	2.62^***^	<10^-5 ns^
3: Ramat Gan (coastal*)	0.30^***^	0.33^**^		0.46^**^	0.62^***^	0.39^***^	0.11^**^	2.60^***^	0.78^***^
4: Nitzanim (coastal*)	0.29^***^	0.22^***^	0.30^**^		1.74^**^	0.60^***^	0.61^**^	2.91^***^	0.70^***^
5: Kishon (Kishon)	0.55^***^	0.56^***^	0.35^***^	0.52^**^		1.4^***^	0.78^***^	1.94^***^	2.03^***^
6: Kinneret (Jordan)	<10^-5 ns^	<10^-5 ns^	0.22^***^	0.27^***^	0.50^***^		0.03^ns^	2.70^***^	0.06^ns^
7: Beit She an (Jordan)	0.10^ns^	0.16^ns^	0.05^**^	0.32^**^	0.40^***^	0.04^ns^		2.35^***^	0.26^**^
8: Ein Feshkha (Dead Sea)	0.87^***^	0.89^***^	0.05^**^	0.84^***^	0.77^***^	0.78^***^	0.75^***^		3.02^***^
9:Ne'ot HaKikar (Dead Sea*)	0.06^ns^	0.04^ns^	0.53^***^	0.52^***^	0.77^***^	0.08^ns^	0.37^**^	1^***^	

Above diagonal: Nei’s corrected average numbers of pairwise differences. Below diagonal: pairwise *Φ*_*st*_ indices. *P < 0.05, **P < 0.01, ***P < 0.001 and ^ns^ non-significant. Introduced populations are indicated by an asterisk.

When including both natural and artificial habitat samples, AMOVA revealed that within-population variation explained about half (48.8 %) of the total genetic variation. The other approximate half of the variation (48.3 %) was explained by variation among populations within the various aquatic systems (*Φ*_*sc*_ = 0.50, *P* < 10^-5^). Variation among aquatic systems was thus small (2.9 %) and non significant (*Φ*_*ct*_ = 0.03, *P* =0.35). Pairwise *Φ*_*st*_ indices as well as Nei’s corrected average numbers of pairwise difference (Table 2) indicated that samples from artificial ponds (Nitzanim and Ramat-Gan) significantly diverged from all other samples and from one another, including those from the coastal system (i.e., the natural system within which the artificial ponds were located). The sample from Ne'ot HaKikar (Dead Sea system) appeared to differ from the second Dead Sea sample (Ein Feshkha) but not from some samples from the Jordan River and coastal water systems. Taken together with its recent discovery, the population of Ne'ot HaKikar is suspected of being a recent introduction. These findings are reflected in the haplotype network (Figure 2).

When the three samples that are presumed to have been artificially introduced (Ne'ot HaKikar, Nitzanim and Ramat-Gan) were excluded from the AMOVA, a significant difference among some of the systems emerged (*Φ*_*ct*_ = 0.58, *P* = 0.013). Analysis of Nei’s corrected average numbers of pairwise differences and pairwise *Φ*_*st*_ indices revealed that the Dead Sea sample of Ein Feshkha differed from the others (2.08 < *D* < 2.61, *P* < 10^-5^). This was also the case for the Kishon River (1.46 < *D* < 2.08, *P* <10^-5^). However, the divergence between the Jordan River and coastal systems was not significant (*D* = 0.02, *P* = 0.25).

### **Meristic counts**

**Figure 3:**
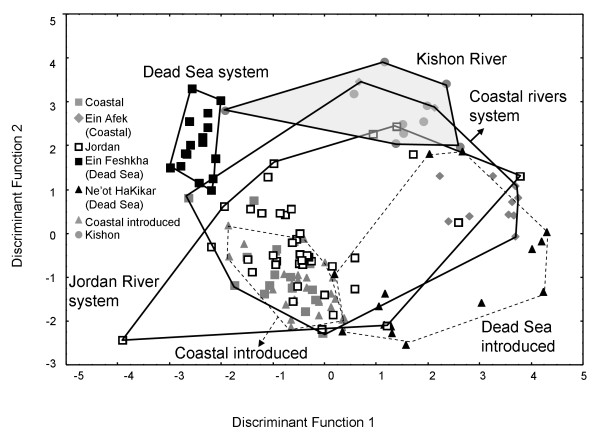
**Discriminant analysis of*T. zillii*meristic counts.** Individuals are from the following groups: gray square, coastal; gray diamond, Ein Afek (coastal); white square, Jordan; black square, Ein Feshkha (Dead-Sea); black triangle, Ne’ot HaKikar (Dead-Sea introduced); gray triangle, coastal introduced; gray circle, Kishon.

In the nested MANOVA of 22 meristic characters (Table 3) the factors "population within aquatic systems" and "aquatic system" significantly partitioned the variance of the meristic counts (F_105_, _538_ = 4.19, *P* < 10^-5^). MANOVA results were not affected by the inclusion or removal of samples from the introduced populations. A scatterplot of the first and second discriminant functions (accounting for 36% and 25.3% of the variation, respectively) clearly separated the Ein Feshkha sample from all others. In this plot (Figure 3), individuals of the coastal sample from Ein Afek and the (probably introduced) sample from Ne'ot HaKikar partly overlap. The highest correct classification rates, according to meristic counts, were of the Ein Feshkha sample (93.8%) and the Kishon sample (90%), while the percentage of correct classification of the other samples ranged from 56 to 83. Two characters were found to influence the first and second discriminant functions, respectively: the position of the longest pectoral ray; and the number of scales between the lower lateral line and the anal fin. The mean values and standard deviation of these characters in each sample are presented in Table 4..

**Table 3:** Meristic characters considered

**Code**	**Meristic count**
LL1	Number of scales along the upper lateral line
LL2	Number of scales along the lower lateral line
TR1	Number of scales between the dorsal fin and the upper lateral line
TR2	Number of scales between the upper and lower lateral line
TR3	Number of scales between the lower lateral line and the anal fin
P	Number of rays in the pectoral fin
Pbr	Number of branched rays in the pectoral fin
V	Number of rays in the ventral fin
Vbr	Number of branched rays in the ventral fin
D	Number of rays in the dorsal fin
Dbr	Number of branched rays in the dorsal fin
DS	Number of spikes in the dorsal fin
A	Number of rays in the anal fin
Abr	Number of branched rays in the anal fin
AS	Number of spikes in the anal fin
C	Number of rays in the caudal fin
Cbr	Number of branched rays in the caudal fin
LPR	The position of the longest pectoral ray
LVR	The position of the longest ventral ray
LDR	The position of the longest dorsal ray
LAR	The position of the longest anal ray
GR	The number of gill rakers

**Table 4:** Sample sizes, mean standard body length and the mean of key meristic counts

**Aquatic system**	**Coastal**					**Kishon**	**Jordan River**		**Dead Sea**	
Sample	Taninim	Ein Afek	Ramat Gan^*^	Nitzanim^*^	HaMa'apil^*^	Kishon	Kinneret	Beit She'an	Ein Feshkha	Ne'ot HaKikar^*^
Data set										
Molecular	13	10	15	10	2	10	16	13	13	15
Meristic	16	12	15	15	0	10	25	14	16	15
SL±sd (cm)	8.5±2.9	4.7±4.3	6.7±2	6.8±4.8		13.2±1.6	10.6±5.2	10.1±2.1	12.3±4.3	4.6±1.4
TR3±sd	6±0.5	6±0.7	6.1±0.3	6.2±0.7		7±0.6	6±0.4	6±0.8	7±0	5±0.6
LPR±sd	4±0	5±0	4±0	4±0		5±0.3	4.2±0.4	3.9±0.3	4±0	4±0.5

* Introduced population, SL = mean standard body length, sd = standard deviation of standard body length. HaMa'apil sample was not measured due to small sample size. TR3 = Number of scales between the lower lateral line and the anal fin. LPR = The position of the longest pectoral ray

## **Discussion**

Based on our results, the geological structure of the examined water systems do not account completely for the population structure of *T. zillii* in Israel. Two natural water bodies, Ein Feshkha (Dead Sea system) and the Kishon River, possess fish populations that are inferred to be isolated from the other water bodies. Conversely, fishes from the two major water systems, the coastal rivers system and that of the Jordan River, could not be differentiated based either on molecular or morphological data.

The distinction of the Kishon River from the rest of the coastal rivers based on its ichthyofauna composition [3] is supported by the molecular and morphological data presented here. However, this difference may also be the result of a recent severe bottleneck as this river has been heavily polluted for several decades [9].

The isolation of the Ein Feshkha Dead Sea system natural population based on molecular and morphological data corresponds to the geological structure of the inland water systems of Israel, where the hypersaline water of the Dead Sea poses a biological barrier for most aquatic organisms between the Jordan River and the Dead Sea system [3], a barrier that has existed for 60,000 years [10]. Isolation by distance and drift are two additional effects that may explain the genetic and morphological divergence of this population, since it is geographically distant from all other sampling sites considered, and one of the smallest water bodies sampled.

The lack of isolation between the coastal rivers' water system and the Jordan River water system could be an artifact of the small sample-size considered in our analysis, which does not allow us to identify significant differences. In particular, it is highly likely that the populations of the Costal system and the Jordan River basin are larger and less affected by drift than the populations present in the small Dead Sea water bodies or the isolated artificial ponds. Another explanation for the lack of isolation between the coastal system and the Jordan River system could be the translocation of *T. zillii* due to ancient aquaculture and trade [11-13].

Concerning the origin of fish present in artificial ponds, the most likely hypothesis is that these fish were introduced when the ponds were populated with fish farm stocks. Although *T. zillii* is not cultivated in Israel [8], it is found in fish farms [14]. Since there are no records of import of *T. zillii*, the individuals present in fish farms are thought to have colonized the fish farm pools during flooding. This is supported by the fact that the haplotypes present in the sampled artificial ponds do not differ from the haplotypes present in the natural water bodies, which suggests a local origin. In the case of the Ne'ot HaKikar (Dead Sea) population, the fact that the first *T. zillii* were spotted in that habitat following the establishment of a fish farm specializing in tilapiine stocks (M. G. personal observation), suggests that the fish might have escaped from the fish farm. This possibility is supported by the fact that another species, *Sarotherodon galilaeus*, is present in Ne'ot HaKikar as a result of its escape from the same fish farm (M. G. personal observation, [15]). The hypothesis of an influence of the fish farm is also supported by the fact that *T. zillii* from Ne'ot HaKikar are more similar both genetically and morphologically to fish from the Ein Afek coastal river population than to those of the Ein Feshkha Dead Sea population.

## **Conclusions**

The mitochondrial and morphological variability of the *T. zillii* population is only partially explained by the geographic distribution of the species in Israel, using the control region sequence as a molecular marker and meristic characters as morphological markers. The possibility that *T. zillii* infiltrates fish farms and might be transported unintentionally together with target species into new environments should be examined in more detail, since such translocation could have an impact on the fish diversity. Future studies based on additional and more variable markers would allow a better resolution of the issues presented here, along with larger sample sizes per location.

### **Availability of supporting data**

The data sets supporting the results of this article are available in GenBank under accession numbers EU163705-EU163723 and FJ613474-FJ613479 and in the Dryad Repository: http://dx.doi.org/10.5061/dryad.29g768sc.

## **Methods**

### **Fish sampling and preparation**

The research was approved by the Israel Nature and Parks Authority (permits 2006/25631, 2007/28858, 2008/31863). Fish were sampled using a seine net. Seven habitats were sampled: a) two coastal system habitats (Ein Afek on the Na'aman River, one sampling site; and the Taninim River, two sampling sites), b) the Kishon River (one sampling site), c) two habitats of the Jordan River system (Lake Kinneret, two sampling sites; and the Beit She'an Valley springs, three sampling sites), d) two Dead Sea system habitats (the Ein Feshkha Nature Reserve, two sampling sites; and the Ne'ot HaKikar Salt Flat, one sampling site). The sampling sites of each water body were at most five kilometers apart. Due to the small sample sizes, data from the same water body were pooled (i.e., they were treated as a single population) in the statistical analyses (AMOVA - Analysis of Molecular Variance and MANOVA - Multivariate Analysis of Variance). Consequently, our design did not allow us to analyze population structure within water bodies, although we cannot exclude the possibility that such a structure exists. We test here only the existence of structure among water basins.

Two artificial recreational ponds were also sampled (the Ramat-Gan park pond and the Nitzanim Nature Reserve pond, Figure 1). These artificial ponds are in the coastal basin. In addition, we obtained two *T. zillii* individuals from the HaMa'apil fish breeding farm. Unfortunately, other breeding farms did not allow us to examine their fish stock. For the morphological analysis, we also included specimens from the Steinhardt National Collection of Natural History, Zoological Museum at Tel-Aviv University (hereafter NCNH). Sample sizes and mean standard body lengths are provided in Table 4.

A liver sample was removed from each specimen collected (Table 4) and fixed in 70% ethanol for DNA extraction. The fish bodies were also preserved in 70% ethanol for meristic counts and submitted to the NCNH. While the individuals sampled were used for both morphological and genetic analysis, we extended our morphological sample size with the addition of fish from the NCNH. Unfortunately, the museum specimens were found to be unsuitable for DNA extraction. Before including these data in our morphological analysis, we verified that the date of capture and methods of conservation were not significant factors, using the non-parametric MANOVA test [16] as implemented in the software PAST [17]. Sample sizes are provided in Table 4. Further sampling would have potentially put the small studied populations at risk.

### **DNA extraction and amplification**

For each individual, 20–40 mg of liver tissue preserved in ethanol was homogenized with 500 μl TNES-Urea buffer [18] and 12.5 μl of proteinase K for 3 hours at 37°C. Following homogenization, DNA was extracted using a standard phenol-chloroform protocol followed by ethanol–sodium acetate precipitation. We selected the mitochondrial control region (D – loop) as our molecular marker, as in other studies of cichlid populations [19-21]. The control region was amplified using the primers Ormt 449 up [22] and a newly designed primer mit-tRNA-phe (5'-AGGGYCYATCTTAACATCTTCAGTG-3'). Amplified fragments were directly sequenced (to avoid cloning artifact) on an ABI PRISM 3100 (Applied Biosystems) genetic analyzer. The fragments were sequenced on both strands. Additionally, each polymorphic site and indel was confirmed manually by looking at the corresponding region on the chromatogram. Haplotypes were submitted to GenBank under accession numbers EU163705-EU163723 and FJ613474-FJ613479. Sequences were aligned using ClustalX [23].The resulting alignment was then refined manually. The final alignment included 862 positions with two indels [Additional file 1.

### **Sequence data analysis**

The software Arlequin 3.0 [24] was used to compute several diversity indices (Table 1): the haplotype diversity *h*[25], the nucleotide diversity *π*[25,26] and two estimators of *θθ*_*s*_, and *θ*_*π*_. *θ* is the effective size of the population multiplied by the mutation rate. The *θ*_*s*_ estimate of *θ* is based on the relationship between the number of segregating sites and the sample size [27], while *θ*_*π*_ is the mean number of pairwise differences in the sample [26]. Both estimators assume the infinite site model. Since the mutation rate for the *T. zillii* control region sequence in unknown, the *θ* estimators can only be used to infer the relative effective sizes of the populations and consequently the relative effect of drift. Arlequin 3.0 was also used to conduct an AMOVA and to compute fixation index estimates (*Φ* estimates) [28]. The total molecular variance was partitioned into four "aquatic systems" (i.e., coastal, Kishon, Jordan River and Dead Sea), "Populations within aquatic system" and "within population" components. The sample sizes did not allow the examination of population structure within water bodies. Two analyses were conducted. In the first, samples from artificial habitats (i.e., Ramat-Gan and Nitzanim) were considered as coastal, since these habitats are situated within the coastal basin, and the Ne'ot HaKikar sample, which has been suspected of having an aquacultural origin, was considered to belong to the Dead Sea system. In the second, samples of populations, which were probably introduced (i.e., Ramat-Gan, Nitzanim and Ne'ot HaKikar) were excluded. The HaMa'apil sample was excluded from the AMOVA analysis due to its small size (n = 2). Pairwise *Φ*_*st*_ values were tested with an exact contingency test as implemented in Arlequin 3.0. When aquatic systems or populations were found to be significantly different, Nei’s corrected average numbers of differences *D*[29] were computed between systems or populations with the program Arlequin, using Jukes-Cantor distances. Finally, a median-joining network [30] was reconstructed with the program Network 4.2.0.1 (available at http://www.fluxus-engineering.com). In all analyses, gaps were treated as a fifth character.

### **Meristic data acquisition and analysis**

Twenty-two meristic characters were counted manually (Table 3). The data were logarithmically transformed to eliminate size-related differences [31]. The meristic counts were analysed using a nested MANOVA to examine whether the variation of morphological traits was significantly partitioned by geographical factors (i.e., among populations and among aquatic systems) discriminant analysis was used to visualize the morphological variation among populations [32-34]. These analyses were conducted with the software Statistica (StatSoft) and PAST. Morphological data were deposited in the Dryad Repository: http://dx.doi.org/10.5061/dryad.29g768sc.

## **Competing interests**

The authors declare that they have no competing interests.

## **Authors' contributions**

AS carried out the field work and morphological analysis under supervision of MG. He performed the sequencing and genetic analysis under the supervision of DH. MG conceived the study. AS and DH wrote the manuscript. All authors read and approved the final manuscript.

## Supplementary Material

Additional file 1 *Tilapia zillii* D-loop sequence alignment. Description: D-loop sequence alignment used in the analysis in Fasta format. TM, Taninim (coastal); EA, Ein Afek (coastal); KN, Kishon (Kishon); KT, Kinneret (Jordan); BS, Beit She’an (Jordan); EF, Ein Feshkha (Dead Sea); NH, Ne’ot HaKikar (Dead-Sea introduced); RG, Ramat Gan (coastal introduced); NM, Nitzanim (coastal introduced); MA, HaMa'apil (coastal introduced). Click here for file
